# New psychoactives within polydrug use trajectories—evidence from a mixed‐method longitudinal study

**DOI:** 10.1111/add.15422

**Published:** 2021-01-28

**Authors:** Kathryn Higgins, Nina O'Neill, Leeanne O'Hara, Julie‐Ann Jordan, Mark McCann, Tara O'Neill, Mike Clarke, Tony O'Neill, Grace Kelly, Anne Campbell

**Affiliations:** ^1^ Centre for Evidence and Social Innovation Queen's University Belfast Belfast UK; ^2^ School of Social Sciences, Education and Social Work Queen's University Belfast Belfast UK; ^3^ School of Nursing and Midwifery Queen's University Belfast Belfast UK; ^4^ IMPACT Research Centre Northern Health and Social Care Trust Antrim UK; ^5^ MRC/CSO Social and Public Health Sciences Unit University of Glasgow Glasgow UK; ^6^ School of Psychology Queen's University Belfast Belfast UK; ^7^ School of Medicine, Dentistry and Biomedical Sciences Queen's University Belfast Belfast UK

**Keywords:** Legal highs, mephedrone, new psychoactive substances, risk, synthetic cannabinoids, taxonomy

## Abstract

**Aims:**

To provide public health‐related research evidence on types and usage patterns of new psychoactive substances (NPS), developmental pathways into NPS and decision‐making factors for, and associated harms of, NPS use.

**Design:**

Three‐phase mixed‐methods design, including a latent class analysis (LCA) of the longitudinal Belfast Youth Development Study (BYDS), a narrative analysis of interviews with NPS users and a three‐step approach manual method modelling using regressions to reveal classes of substance use and their associated predictors and outcomes.

**Setting:**

Northern Ireland.

**Participants:**

A total of 2039 people who responded to the questions on ‘ever use’ of the drug variables included at wave 7 (aged 21 years) of the BYDS. Eighty‐four narrative interviews with NPS users.

**Measurements:**

Categories of drug use identified by LCA. Predictors and outcomes included measures of family, partners, peers, substance use, school, delinquency and mental health.

**Findings:**

A four‐class solution provided the best fit for the data: alcohol; alcohol and tobacco; alcohol, tobacco and cannabis; and polydrug (the latter including NPS). The qualitative analysis yielded a taxonomy that distinguished how NPS operate within a wider range of drug repertoires from experimental to problematic.

**Conclusions:**

In Northern Ireland, new psychoactive substances appear to be a feature of broader polydrug use rather than a standalone class of drug use.

## Introduction

The use of multiple substances or ‘polydrug’ use is an increasing phenomenon [1, 2, 3, 4]. However, what is denoted by ‘polydrug use’ is not agreed upon, either in terms of substances included or intensity of use. Conceptually, polydrug use encompasses wide variation in user populations and in patterns of use. Historically, the literature distinguished between socially marginalized users of heroin and a range of other substances, such as cocaine, benzodiazepines and alcohol, and socially integrated people using combinations such as ‘cocaine and alcohol’ or ‘cannabis and alcohol’ problematically [1, 5]. Also reflected are two broad types of polydrug use: ‘concurrent’, referring to the use of more than one drug during a given period; and ‘simultaneous’, meaning the use of two or more substances on the same occasion [6, 7]. Changes in chemical and multi‐media technologies have significantly increased production, availability and accessibility of drugs [8], making the use of multiple substances a more common occurrence, with previous distinctions blurred [2, 3, 4]. New psychoactive substances (NPS) are a major contributor to this complex and changing polydrug vista, presenting a global challenge in terms of public health impact [5, 9]. This challenge is further exacerbated by the lag between empirical evidence and evolving consumption practices. For example, knowledge concerning patterns of multiple substance use that include NPS is limited, particularly in terms of overlap across different drug‐using populations [3]. NPS formerly, although inaccurately, known as ‘legal highs’ (NPS were never legal for human consumption), is the name given to drugs that are newly synthesized or newly available and which do not fall under the control of the 1961 Single Convention on Narcotic Drugs or the 1971 Convention on Psychotropic Substances, but which may potentially be harmful [9]. The definition also includes counterfeit medications, designed to mimic the effects of existing substances. During the past decade, NPS have increasingly featured as a major topic of discussion in policy and research documents [3, 10, 11] and these substances have been appearing on the drugs market at a rapid rate [5].

Potentially, the emergence of an NPS market could interact with the traditional drug market in a number of ways: (1) there could be a substitution, where NPS replaces previous traditional drugs as they are preferred in terms of price or effect. (2) They could contribute to the mixed‐use pattern, with people who use multiple drugs selecting equally from traditional drugs and NPS. (3) In the context of dependency, NPS may occupy a specific market segment, being used primarily among certain populations (e.g. the most marginalized). Each of these potential patterns may have a different corresponding policy response in terms of supply disruption, preventive health or treatment provision, hence this study aims to understand which structure or structures have emerged.

This paper reports on a mixed‐method study investigating NPS in the context of this increasingly dynamic drug scene. We aimed to: (1) identify types and patterns of NPS use and how they relate to other substance use; (2) examine the developmental pathways into NPS use and specific types of NPS; (3) identify key risk and protective factors for substance use; (4) test for associations between NPS use and health and social outcomes; and (5) provide evidence on NPS to inform existing service provision/policy formation and educational initiatives. Further specific research questions are covered in detail in the full report [12].

## Methods

### Design

The study leveraged data from the Belfast Youth Development Study (BYDS) to create a three‐phase mixed‐methods design with a shared conceptual framework. BYDS is an 18‐year longitudinal cohort study investigating substance use behaviours from early adolescence to adulthood. It has seven waves of data (collected between 2001 and 2011) when participants were aged 11/12–20/21 years. In addition to data on substance use, BYDS contains a rich source of information on issues relevant to this age cohort, including parent–child attachment, parental monitoring and household composition, school attachment and connectedness, peer and friendship networks, education, training, employment, physical and mental health, pro‐ and anti‐social behaviour and the formation of romantic experiences. The analysis plan was not pre‐registered and these results should be considered exploratory.

Phase 1 involved secondary analysis of BYDS to identify categories of drug use and how NPS relates to use of other substances. This informed the sampling framework for the recruitment of participants for Phase 2, which included 84 narrative interviews. This cohort encompassed BYDS participants (*n* = 25), those in contact with drug and alcohol services (*n* = 34) and individuals currently incarcerated across three prison sites in Northern Ireland (NI) (*n* = 25). Eligibility criteria was self‐reported life‐time use of any NPS (defined by responses to two of the drug use questions in the BYDS: ‘Have you ever used mephedrone?’ and ‘Do you use any drugs called legal highs?’). Ethical approval was granted by Queen's University Belfast (QUB) Research Committee, Office for Research Ethics Committees NI (ORECNI) and the NI Prison Service. Interviews were conducted in private offices in QUB, community centres and, for those incarcerated, interviews took place in the same space used for consultations between prisoners and keyworkers. Interviews commenced in April 2016 and ceased in June 2017. In Phase 3, the BYDS data set was used to examine early risk and protective factors associated with membership of specific classes of drug use. With the majority of the interview sample having been recruited from services and prisons, it is acknowledged that interview data largely depict more problematic patterns of substance use.

## Analysis

### Phase 1: latent class analysis (LCA)

Patterns of substance use were investigated by conducting an LCA on the responses to ‘ever use’ drug‐related questions answered by 2039 BYDS participants who participated at age 21 years (wave 7). The substance use questions submitted to the analysis covered a broad range of substance types, including alcohol, tobacco, cannabis, other pills, 1 ecstasy, speed, lysergic acid diethylamide (LSD), poppers, cocaine, NPS and heroin. The LCA was run in MPlus version 7 with maximum likelihood estimation, 100 iterations and randomly generated start values. The indicators included in the model all had low levels of missingness (< 0.5%); missingness on these indicators was handled via the maximum likelihood estimation procedure. Model fit was judged via a range of fit statistics; namely, Akaike information criterion (AIC); Bayesian information criterion (BIC); sample‐size adjusted Bayesian information criterion (ABIC); entropy; and the Lo–Mendell–Rubin likelihood ratio (LMR) and bootstrapped Lo–Mendell–Rubin likelihood ratio tests (BLMR). The LCA was repeated five times to test and compare fit of models with two to six classes. From these LCAs models with three and four classes emerged as having the best fit. After inspecting the respective plots for these models, it became clear that the four‐class solution provided the clearest differentiation between the classes, and hence the four‐class solution was adopted. As a sensitivity analysis, we ran the LCA excluding respondents who reported never using drugs apart from alcohol and tobacco. This model did not uncover a unique NPS class, and the substantive interpretation of the model was the same as for the main model (see Supporting information, S1).

### Phase 2: narrative analysis

The definition of NPS used emerged organically during interviews. The most commonly reported NPS were SCs and mephedrone. Less common, but also mentioned, were herbal pills, China white, other stimulant NPS and hallucinogen NPS. Narrative accounts were used to explore trajectories of NPS use utilizing a five‐code type framework [13]. A four‐group taxonomy was constructed from key conceptual fields. These included broad risk and protective factors as articulated by participants at individual, family and community level, detailed substance use repertoires and location of NPS therein, patterns of use, primary motivations for substances used and critical incidents that either resulted in desistance or increased use. Identifying key conceptual domains and significant dimensions within those domains provided the analytical framework (for greater detail see Chapter 2, pages 15–16 of the main report [12]). For example, participants’ viewpoint on NPS was explored alongside reported risk and protective factors from childhood to the present day. We examined how participants perceived that risk and their patterns of drug use/risk behaviour were recorded. This structure accommodated both the inductive and deductive components of the study. Analyses were conducted in NVivo version 11. The team (quantitative and qualitative) met to review any discrepancies and resolved minor differences by negotiated consensus.

### Phase 3: risk factors and outcomes analysis

The shared risk factor of the model was tested using the three‐step approach manual method [14]. This involved using the BYDS longitudinal data file to examine early risk and protective factors associated with latent class membership at age 21 years (reference group: polydrug users). Eight broad types of predictors were included in the models. These were family, peers, substance use history, school, delinquency, mental health/personality, partner and demographics. Initial status and growth rates were used for longitudinal variables (see Supporting information, Fig. S1, Tables S3 and S4 for calculation details). Following this, outcomes associated with prior life‐course drug history were examined using linear and logistic regression models while controlling for class membership probabilities (reference group: polydrug users), as well as confounding variables. There were no missing values on the latent class membership variable; missingness rates on the predictor (1–63%) and outcome variables (2–20%) varied depending on the survey wave at which they were measured. Multiple imputation was used to deal with missing data in the risk and outcome factors analyses, and extensive sensitivity tests were used to evaluate the quality of the imputation models.

Several integration analyses were built into the design to maximize the utility of the sequential mixed‐methods design (see Supporting information, Fig. S2). Specifically, integrating quantitative material with qualitative information allowed emerging findings to be further explored and cross‐validated and later phase methodologies to be refined.

## Results

### Identifying where NPS use sits within substance use classes (LCA)

**Figure 1:**
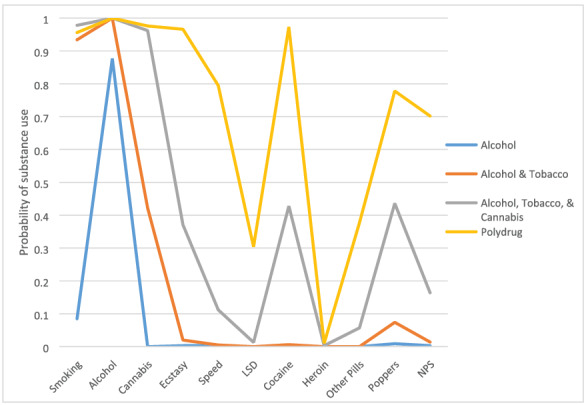
Probability of substance use in each of the four latent classes identified in the Belfast Youth Development Study (BYDS) data

LCA suggested that there was not a distinctive ‘NPS class’ among our sample and identified four broad types (latent classes) of substance use: alcohol, tobacco and cannabis (ATC, *n* = 367: 18%), alcohol and tobacco (AT; *n* = 926; 45%), alcohol (A; *n* = 532; 26%) and polydrug use (*n* = 214; 10%). Classes were named according to the substances for which the probability of previous use was 50% or above (see Fig. 1). For the polydrug class, the LCA revealed probability at or above 50% for previous use of alcohol, tobacco, cannabis, ecstasy, speed, cocaine, poppers and NPS and below 50% for all other substances. There was no evidence for a unique ‘NPS class’; NPS use is a feature of broader polydrug use. In fact, among polydrug users in our sample, 71% reported previous NPS use.

### Narrative interviews

Motives, characteristics and lived experiences of participants using NPS were explored to categorize the data into meaningful types. Four main groups emerged: these ranged from ‘experimental’ type users whose experience was mainly limited to alcohol and cannabis, to the ‘dependents’ who needed help from health and social care services for addiction to NPS and/or other substances. The ‘dependents’ were the largest group and varied significantly. This group was further subdivided into four smaller groups: these ranged from the ‘generation’ where NPS were the primary and only dependency to those who were ‘non‐NPS’ users. See Table 1 for details, including key demographics and the sampling source.

**Table 1:** Four‐group taxonomy.

Sample group	Group name	*n*	Gender	Age range	Mean age	Source
						BYDS
						service user (SU)
			Male (M)			
						Her Majesty's Prison (HMP)
			Female (F)			
Group 1	Limited experimentals	8	M (3)	19–27	24	BYDS (5)
						SU (3)
						HMP (0)
			F (5)			
Group 2	Past recreationals	14	M (5)	17–28	26	BYDS (11)
						SU (3)
						HMP (0)
			F (9)			
Group 3	Contemporary regulars	10	M (5)	16–28	25	BYDS (7)
						SU (2)
			F (5)			
						HMP (1)
Group 4	Dependents	52	M (44)	17–56	28	BYDS (2)
						SU (26)
						HMP (24)
			F (8)			
Subgroup 1	Generation NPS	14	M (11)	17–24	19	BYDS (0)
						SU (9)
						HMP (5)
			F (3)			
Subgroup 2	Availers	15	M (14)	19–50	32	BYDS (2)
						SU (6)
						HMP (7)
			F (1)			
Subgroup 3	Persistors	18	M (14)	18–53	28	0/9/9
			F (4)			
Subgroup 4	Non‐NPS users	5	M (5)	34–56	42	0/2/3
			F (0)			

BYDS = Belfast Youth Development Study.

For group 1, the ‘limited experimentals’ NPS did not feature in the groups’ drug storyline. The decision not to use NPS was a conscious one, partly motivated by the existence of more protective factors and fewer risk factors than other participants. The ‘experimentals’ had reasonable knowledge about NPS and displayed an awareness of the level of ‘unknowns’ associated with use, which contributed to other disincentives for use.

The ‘past recreationals’ were characterized by the use of largely stimulant type NPS (e.g. mephedrone) within a pattern of alcohol, cannabis, cocaine and other stimulant type substances. Legal status, low cost and easy accessibility featured significantly as a lure towards use, which tended to be recreational and opportunistic. The use of synthetic cannabinoids (SCs) was mentioned infrequently and, in contrast to mephedrone, use was usually an isolated incident. Participants demonstrated good knowledge of the pharmacological properties of various NPS. Like the ‘experimentals’, knowledge acted as a protective factor.

The ‘contemporary regulars’ use of NPS (mainly mephedrone) sat within a very experimental profile and included ketamine and gamma hydroxybutyrate (GHB), as well as stimulant NPS beyond mephedrone. In line with other research, use of substances was significant for achieving personal goals in early adulthood; for example, retaining social networks [15] and finding romantic partners [16]. In contrast to groups 1 and 2, it was not legal status that featured as an incentive for use, but market forces. Significant here were references to the perceived reduced quality of cocaine compared to the high quality of mephedrone. The interplay between risk and protective factors was complex. For example, while positive parenting acted as a protective factor and lack thereof posed a risk for groups 1 and 2, responsible parenting practices were more likely to result in clandestine drug use behaviour as opposed to lessening drug‐taking, as reported by others [17].

For the ‘contemporary regulars’, knowledge of NPS tended to be channelled into maximizing effect and adopting harm‐reduction strategies as opposed to limiting use.

The ‘dependents’ were overwhelmingly male, with typically more chaotic life stories. This is also reflected in the source of sampling—of the 52 people in the ‘dependents’ group, 26 participants were in contact with drug and alcohol service providers and 24 were imprisoned. First initiation mainly involved alcohol, but for some it was cannabis or multiple substances. While all participants in group four reported issues with dependency, patterns and intensity of use varied significantly. To aid analysis, the ‘dependents’ were subdivided into four groups.

For subgroup 1 (SG1) ‘generation NPS’, NPS represented the primary and only dependency, while still within a polydrug use trajectory. They were the closest group to an identified distinct NPS class. Notable in this group, however, was the rapid progression for some from alcohol use to dependence on SCs. Prominent in their narratives were the use of alcohol, cannabis and prescription medication such as diazepam and pregabalin. Significant risk factors were early disengagement with school, lack of parental control, negative peer influence and individual characteristics [e.g. attention deficit hyperactive disorder (ADHD), poor mental health]. They were the youngest of the subgroups, lacked user knowledge and were more likely to be influenced by perceptions of legality, low cost of NPS and easy access. One 17‐year‐old male from this group stated:

When I was 15 that was when I basically got into legal highs—it was cheaper, stronger and you got way more for your money (1046).

In contrast, subgroup 2 (SG2), ‘the availers’, reported dependency on traditional illicit substances, tending to ‘avail’ of NPS only when their drug of choice was not accessible. Knowledge of NPS for SG2 acted as a safety mechanism in terms of mitigating harm by testing out dosage and potency. Subgroup 3 (SG3), the ‘persistors’, reported multiple dependencies, including NPS (mainly SCs and mephedrone). In contrast to SG2, there was a lack of awareness/concern regarding potency and predictability of NPS. One 42‐year‐old female from this group demonstrated this point when she discussed her experience with various NPS:

China White, Dust to Dawn, it melted me right out, melted the head off me, when you were sniffing it after a while it just started melting me like proper melt, I do not know what was in it, it was meant to be like the Speed effect but I do not know what was in it but it melted me clean out, then I tried herbal E, Triple X, it was purple, flip me I was shaking it for four days, it hit me in ten minutes but see the next day for about three days I was shaking and all bad (1019).

Although the ‘persistors’ reported multiple dependencies, SCs often became their drug of choice and in some cases replaced use of heroin.

Subgroup 4 (SG4) were ‘non‐NPS users’ who were dependent upon other substances and were disinterested in NPS. The authors contend that being older than the other subgroups attributed significantly to their indifference to NPS, perceiving it to be a part of growing up, a temptation for the ‘younger generation’. As one SG4 participant explained: ‘We remember what it was like being offered a cigarette, don't we?’. SG4 shared commonalities with the other subgroups in terms of circumstances promoting drug dependency (e.g. poor mental health, negative family environment/relationships). Their age and disinclination to NPS were the main factors which distinguished SG4 from the other subgroups, suggesting that the adverse life situations they shared was not attributed to NPS as much as it was to their existing drug dependency.

### Key risk and protective factors for substance use

Variables were selected from the BYDS data set to align with the risk and protective factors related to substance use that emerged from the qualitative analysis. Risk and protective factor variables were entered into the model in eight blocks (see Table 2). Little distinguished the ATC and polydrug groups, except that the ATC group were less likely to have a partner at age 17 years who used cocaine. Compared to polydrug users, AT users were less likely to have friends at age 17 years who took cocaine or ecstasy, less likely to be in trouble with the police by age 14 years and had less of a reduction in school commitment and going to the park during adolescence. Relative to polydrug users, alcohol users were less likely to have friends who took cannabis or ecstasy when they were aged 17 years, had higher levels of school commitment at age 13 years and lower levels of decline in school commitment over time. This adds nuance to existing evidence from studies highlighting links between sustained engagement with drug‐using peers into late adolescence and the relative contribution that poses for individuals expanding their own polydrug use [18, 19].

**Table 2:** Alcohol, AT and ATC groups compared with polydrug group (reference) on the predictor variables (odds ratios).

	Odds ratios (95% confidence intervals)		
	Alcohol	AT	ATC
Dead	0.32 (0.09, 1.15)	0.71 (0.28, 1.84)	9.57 (0.15, 2.22)
Parent moved	0.64 (0.19, 2.15)	0.90 (0.31, 2.59)	9.59 (0.19, 1.88)
Left home early	0.66 (0.21, 2.09)	0.62 (0.18, 2.09)	9.99 (0.23, 4.20)
Parental drinking view	0.70 (0.47, 1.06)	1.22 (0.84, 1.76)	9.81 (0.53, 1.24)
Parental control intercept	1.02 (0.91, 1.14)	0.99 (0.89, 1.11)	1.01 (0.89, 1.15)
Peer used cannabis	0.17 (0.06, 0.51)^**^	0.42 (0.15, 1.19)	1.77 (0.54, 5.82)
Peer used ecstasy	0.29 (0.10, 0.86)^*^	0.34 (0.13, 0.89)^*^	9.40 (0.14, 1.18)
Peer used cocaine	0.48 (0.18, 1.34)	0.38 (0.17, 0.87)^*^	9.61 (0.26, 1.45)
Bully	1.38 (0.54, 3.49)	1.41 (0.61, 3.25)	1.65 (0.65, 4.21)
Early alcohol use	0.16 (0.00, 5.94)	0.71 (0.31, 1.63)	9.56 (0.24, 1.32)
Early cannabis use	0.44 (0.16, 1.24)	0.50 (0.24, 1.05)	9.72 (0.30, 1.70)
Sought help	2.76 (0.22, 34.75)	1.14 (0.35, 3.72)	9.82 (0.09, 7.11)
School attachment intercept	1.02 (0.92, 1.12)	1.00 (0.90, 1.10)	9.97 (0.86, 1.09)
School commitment intercept	1.50 (1.22, 1.84)^***^	1.13 (0.98, 1.31)	1.13 (0.95, 1.35)
School commitment slope	1.57 (1.24, 2.00)^***^	1.20 (1.01, 1.42)^*^	1.17 (0.96, 1.43)
GCSE 6+	0.67 (0.36, 1.24)	0.91 (0.54, 1.55)	1.33 (0.64, 2.76)
Delinquency	0.86 (0.73, 1.02)	0.90 (0.78, 1.03)	9.97 (0.84, 1.13)
Trouble with police	0.57 (0.26, 1.24)	0.46 (0.24, 0.88)^*^	9.78 (0.39, 1.58)
Street intercept	0.96 (0.65, 1.43)	1.23 (0.84, 1.80)	1.47 (0.93, 2.31)
Street slope	0.57 (0.20, 1.61)	0.49 (0.18, 1.33)	9.63 (0.21, 1.91)
Park intercept	0.86 (0.51, 1.44)	1.12 (0.70, 1.78)	1.07 (0.66, 1.73)
Park slope	2.38 (0.77, 7.29)	3.16 (1.21, 8.23)^*^	2.66 (0.88, 8.03)
Emotional difficulties	1.07 (0.92, 1.24)	1.04 (0.91, 1.18)	1.03 (0.89, 1.20)
Impulsivity	1.00 (0.95, 1.05)	1.01 (0.97, 1.06)	1.02 (0.97, 1.08)
Partner used cannabis	0.35 (0.11, 1.15)	0.46 (0.20, 1.09)	9.84 (0.33, 2.15)
Partner used ecstasy	0.86 (0.19, 3.92)	1.19 (0.35, 4.08)	9.67 (0.13, 3.42)
Partner used cocaine	0.57 (0.10, 3.12)	0.30 (0.09, 1.07)	9.16 (0.03, 0.91)^*^
Gender	1.22 (0.69, 2.15)	1.71 (0.98, 2.98)	1.72 (0.92, 3.24)
Free school meals	1.15 (0.46, 2.86)	1.05 (0.58, 1.90)	1.41 (0.64, 3.09)
Child/children	0.47 (0.15, 1.44)	1.62 (0.73, 3.61)	9.82 (0.31, 2.17)

Estimates are odds ratios.

^*^ *P* < 0.05,

^**^ *P* < 0.01,

^***^ *P* < 0.001.

95% confidence intervals are in parentheses. See Appendix 5 (Table 16) of the main report [12] for comprehensive details on the definition and construction of predictor variables. AT = alcohol and tobacco; ATC = alcohol, tobacco and cannabis; GCSE = general certificate of secondary education.

### Adult outcomes associated with substance use

Analyses controlled for gender, socio‐economic status and the early and protective factors associated with substance use (as per Table 2) in all outcome models. Table 3 presents a condensed version of the results.

**Table 3:** Summary of adjusted regressions for drug class association with outcome variables.

Outcome	Alcohol		AT		ATC	
	Odds ratio (95% confidence intervals)	Sign. (*P*)	Odds ratio (95% confidence intervals)	Sign. (*P*)	Odds ratio	Sign. (*P*)
Psychosis	0.93 (0.88, 0.98)	0.006	0.95 (0.91, 0.98)	0.006	0.99 (0.93, 1.05)	0.814
Self‐harm	0.90 (0.83, 0.97)	0.009	0.93 (0.89, 0.98)	0.009	0.99 (0.89, 1.08)	0.781
Medication	1.01 (0.95, 1.06)	0.762	0.94 (0.90, 0.98)	0.002	1.05 (0.99, 1.12)	0.096
Services	0.99 (0.96, 1.03)	0.724	0.97 (0.95, 1.00)	0.039	1.02 (0.97, 1.07)	0.440
Justice	0.92 (0.86, 0.98)	0.017	0.92 (0.87, 0.97)	0.004	0.97 (0.90, 1.04)	0.427
NEET	0.98 (0.93, 1.04)	0.596	0.99 (0.96, 1.03)	0.676	0.97 (0.90, 1.03)	0.331

	Coeff.	Sign. (*P*)	Coeff.	Sign. (*P*)	Coeff.	Sign. (*P*)
Depression	−0.09 (−0.16, −0.02)	0.013	−0.08 (−0.13, −0.03)	0.002	0.07 (−0.02, 0.15)	0.128
Excessive drinking	−0.34 (−0.44, −0.23)	0.000	−0.21 (−0.27, −0.14)	0.000	0.03 (−0.11, 0.18)	0.643
Drug abuse	−0.12 (−0.15, −0.09)	0.000	−0.09 (−0.12, −0.07)	0.000	−0.11 (−0.16, −0.07)	0.000
Cannabis abuse	−0.14 (−0.19, −0.10)	0.000	−0.11 (−0.15, −0.08)	0.000	−0.14 (−0.20, −0.08)	0.000
Offending	−0.05 (−0.07, −0.03)	0.000	−0.04 (−0.05, −0.03)	0.000	−0.03 (−0.06, −0.01)	0.022

NEET = not in education, employment or training; AT = alcohol and tobacco; ATC = alcohol, tobacco and cannabis. Polydrug group = reference group. See Appendix 5 (Table 17) of the main report [12] for comprehensive details on the definition and construction of outcome variables.

Compared to polydrug users, the AT group were less likely to have poor outcomes in adulthood in most of the areas examined except for being not in education, employment or training (NEET), where outcomes were similar. Similarly, the alcohol group tended to have better adult outcomes than polydrug users, apart from on the variables NEET and use of medication or services for emotional or behavioural problems measures. ATC users were less likely to have poorer outcomes than polydrug users in only a few areas. Specifically, these were drug use, cannabis use and offending.

## Discussion

This study supports the view that NPS may be considered as having a place within a polydrug use path, rather than as a stand‐alone substance type. Although quantitative analysis did not uncover evidence for a distinct class of NPS users, it was possible to differentiate within the overall NPS/polydrug use. Our study has confirmed existing evidence on health and social harms associated with NPS use (particularly mephedrone and SCs). Analysis throughout taxonomy groups provided unique insight of NPS use that augments the current literature. For example, the ‘availers’ would have used both stimulant type NPS and SCs only when their drug of choice was not available. In contrast, although the ‘persistors’ had multiple dependencies, SCs became their drug of choice once they started using them, even in place of heroin.

Generally, dependence tends to be associated with longer and more intense histories of drug use; for example, in the case of heroin users. However, within a polydrug use pathway the data indicate that, in some instances, specific NPS operated as a ‘snare’ to more problematic patterns of use. This was notable in the case of generation NPS (SG1), where SCs played a major role in accelerating pathways to dependency. For some people in SG1, a trajectory from having only used alcohol to dependence on SCs was noticeable.

The evidence presented here points to the volatility of NPS and its unpredictable nature. This was demonstrated by the fact that while sophisticated knowledge of strengths and harms associated with NPS was present (particularly with the ‘contemporary regulars’ and the ‘dependents’), at the same time a number of established users expressed surprise at the strength and unintended effects of SCs and had difficulty anticipating the increasing complexity of SCs. That said, it is noteworthy that the absence of NPS use by SG4 contributed little to modifying the group members existing problems. Rather, it was problematic drug use *per se* that was steering those adverse life experiences [see Chapter 3 of the main report [12] for a comprehensive overview of substances mentioned in the qualitative interviews)].

Although the literature on NPS is in the early stages and lacks data to directly inform an evidence‐informed public health response to NPS [20], the evidence base is increasing. Findings point to the need for a more fine‐tuned approach, tailored to the severity of substance use itself, including NPS, and paying heed to the circumstances that render some people more vulnerable to problematic drug use. This study's results concur with clinical guidance emerging from the available research evidence (see Project Neptune [21]) that interventions take into account the service user's health and other concomitant variables when planning treatment of care. For example, Project Neptune highlights that a substantial proportion of NPS use problems and behaviours were similar to those indicative of stimulant use and alcohol use. Similarly, for the ‘dependents’, NPS usage was couched within a multi‐substance use framework, indicating the importance of considering this when designing treatment modality. Thus, it might be more appropriate to seek to refine existing treatments rather than designing explicit therapies which focus solely on addressing harms associated with NPS. As noted by others, the outcome of wider societal problems often present as problematic use of whatever substance is at hand [8].

The taxonomy links individual characteristics, together with risk and protective factors, alongside distinguishable drug repertoires. This provides a framework that may be used to match effective evidence‐based interventions against different versions of NPS use. The next stage of this research will identify how best to mould these interventions to individual circumstances; for example, targeting declining school engagement or peer substance use (see Table 2), while bearing in mind the capacity of ‘conventional’ risk and protective factors to act in unanticipated ways. A main aim will be the avoidance of movement from lower to higher level categories of use. This is important against a background of increasingly diverse and changing needs which require more complex interventions that can accommodate deviation while operating within structured parameters.

## Declaration of interests

M.C. is currently a member of the NIHR HTA General Board.

## Author contributions

**Kathryn Higgins:** Conceptualization; formal analysis; funding acquisition; investigation; methodology; project administration; resources; software; supervision; validation; visualization. **Nina O'Neill:** Conceptualization; data curation; formal analysis; funding acquisition; investigation; methodology; project administration. **Leeanne O'Hara:** Conceptualization; data curation; formal analysis; funding acquisition; investigation; methodology. **Julie‐Ann Jordan:** Conceptualization; data curation; formal analysis; funding acquisition; investigation; methodology; validation; visualization. **Mark McCann:** Conceptualization; data curation; formal analysis; funding acquisition; investigation; methodology; validation; visualization. **Tara O'Neill:** Conceptualization; funding acquisition; methodology. **Mike Clarke:** Conceptualization; formal analysis; funding acquisition; methodology; validation. **Tony O'Neill:** Conceptualization; methodology; validation. **Grace Kelly:** Conceptualization; data curation; formal analysis; methodology; validation; visualization. **Anne Campbell:** Conceptualization; funding acquisition; methodology.

## Supporting information


**Table S1** Fit statistics for the models with 1–6 clusters (including respondents who reported never using drugs apart from alcohol and tobacco)
**Table S2** Fit statistics for the models with 1–6 clusters (excluding respondents who reported never using drugs apart from alcohol and tobacco)
**Fig. S1** Observed scores on longitudinal predictors throughout adolescence
**Table S3** Fit indices for growth models
**Table S4** Baseline models for BYDS longitudinal variables
**Fig. S2** Design of study.

## Footnotes

^1^
Other pills were described in the questionnaire as ‘Valium, diazepam, jellies, wobbly eggs, tamazepam’. This was followed by the following instruction: ‘Do not count tablets you take when you are ill or that are given to you by your doctor’.
