# Supplementary material for: New psychoactives within polydrug use trajectories—evidence from a mixed‐method longitudinal study
Source: Addiction. 2021 Jan 28;116(9):2454–62. doi: 10.1111/add.15422 (PMC8638670; doi:10.1111/add.15422)
Supplement: Supplementary file 1 — Table S1 Fit statistics for the models with 1–6 clusters (including respondents who reported never using drugs apart from alcohol and tobacco) Table S2 Fit statistics for the models with 1–6 clusters (excluding respondents who reported never using drugs apart from alcohol and tobacco) Fig. S1 Observed scores on longitudinal predictors throughout adolescence Table S3 Fit indices for growth models Table S4 Baseline models for BYDS longitudinal variables Fig. S2 Design of study. [file ADD-116-2454-s001.docx]

**Supporting Information**

**S1 - Four class solution development**

**Sensitivity analysis**

As a sensitivity analysis, we ran the Latent Class Analysis excluding respondents who reported never using drugs apart from alcohol and tobacco. The results with these respondents included and excluded are presented in Tables S1 and S2. The model excluding respondents who reported never using drugs apart from alcohol and tobacco did not uncover a unique NPS class, and the substantive interpretation of the model was the same as for the main model (see Appendix).

As shown in Table S2, the three, five and six class solutions were favoured by the BIC, AIC, and SSABIC respectively. Only the 2 class and 5 class solution had an entropy above 0.8 and classification probabilities were poor for the 3 and 4 class solution. Hence, we focus our substantive interpretation on the 2 and 5 class solution.

The 5 class solution contain classes that were subdivisions of cannabis users (cannabis only, and cannabis + poppers), and subdivisions of polydrug users. All of the polydrug classes reported NPS use, the subdivisions identified groups not using cannabis, or not using LSD as part of the polydrug use repertoire. (General polydrug, club drug (pills + powder + NPS) but not cannabis, polydrug but not LSD).

However, one of the classes was very small (< 5%), there were quite a few boundary values within some of the classes, and one of the classes had a classification probability of .77 which was slightly short of the desirable level .

Table S1: Fit statistics for the models with 1-6 clusters (including respondents who reported never using drugs apart from alcohol and tobacco)

| No. of classes | Loglike-  lihood | AIC | BIC | SSABIC | LMR-LRT (*p*) | BS-LRT | Entropy |
| --- | --- | --- | --- | --- | --- | --- | --- |
| 1 | -7793.80 | 15609.59 | 15671.41 | 15636.46 | N/A |  | N/A |
| 2 | -6039.21 | 12124.41 | 12253.68 | 12180.60 | 3471.22  (*p* < .001) | 3509.18  (*p* < .001) | 0.89 |
| 3 | -5674.01 | 11418.02 | 11614.72 | 11503.53 | 722.49  (*p* < .001) | 730.39  (*p* < .001) | 0.83 |
| 4 | -5612.82 | 11319.64 | 11583.79 | 11434.47 | 121.05  (*p* < .001) | 122.37  (*p* < .001) | 0.77 |
| 5 | -5588.16 | 11294.32 | 11625.91 | 11438.46 | 48.79  (*p* = .010) | 49.33  (*p* <.001) | 0.74 |
| 6 | -5576.52 | 11295.03 | 11694.07 | 11468.49 | 23.04  (*p* = .169) | 23.29  (p = .143) | 0.76 |

Table S2: Fit statistics for the models with 1-6 clusters (excluding respondents who reported never using drugs apart from alcohol and tobacco)

| No. of classes | Loglike-  lihood | AIC | BIC | SSABIC | LMR-LRT (*p*) | BS-LRT | Entropy |
| --- | --- | --- | --- | --- | --- | --- | --- |
| 1 | -3892.56 | 7803.11 | 7847.50 | 7818.91 | N/A |  | N/A |
| 2 | -3198.84 | 6435.68 | 6529.37 | 6469.03 | 1367.71  (p < .001) | 1387.44, (p < .001) | 0.874 |
| 3 | -3152.94 | 6363.87 | 6506.88 | 6414.78 | 90.50  (p <.001) | 91.80  (p < .001) | 0.698 |
| 4 | -3123.54 | 6325.08 | 6517.41 | 6393.54 | 57.95  (p <.001) | 58.79  (p < .001) | 0.774 |
| 5 | -3103.16 | 6304.32 | 6545.96 | 6390.33 | 40.19  (p = .001) | 40.77  (p < .001) | 0.842 |
| 6 | -3089.07 | 6296.15 | 6587.11 | 6399.72 | 27.77  (p = .267) | 28.17  (p = .032) | 0.802 |

**Figure S1, Table S3, Table S4**

**Deriving initial status and growth measures for the longitudinal risk factors**

Several of the variables selected for the quantitative model were continuous and measured at 4 or more waves (w): school attachment w2-5; school commitment w2-5; parental control w2-5; street w1-5 and park w1-5. Intercepts (initial status) and slopes were computed for these variables using growth curve modelling (GCM) which applies structural equation modelling methodology to the analysis of longitudinal data. During GCM growth trajectories are fit to observed data (Figure S1), and any deviations of the observed scores from the trajectory are considered to represent measurement error. The models were fit using maximum likelihood with robust standard errors (MLR).

As per Bollen and Curran’s (1) recommendations, a variety of fit indices were used to assess the models (Table S3). These indices suggest excellent fit for the school attachment, parental control and park models. For the other models (school commitment and street), all indices except the chi square test suggest excellent model fit. Of course, in large samples where there is an excess of statistical power, chi square tests often lead to a rejection of the null hypothesis (1). Where this occurs it is recommended to judge model fit using a variety of indices that are not sensitive to sample size (e.g. Comparative Fit Index (CFI), Tucker-Lexis Index (TLI) and Root-mean-square error of approximation (RMSEA)); these particular indices suggest that the school commitment and street models have excellent model fit. For the school attachment, parental control, street and park models quadratic models were fit. By contrast, for school commitment, a freed loading model provided the best fit.

Table S4 shows the mean intercept, slope and quadratic parameters, alongside the corresponding variances for each model. The intercept and slope variances were significant for all models, this excludes the parental control model for which only the intercept variance was significant. Due to non-significant variation in the slope variance for parental control, parental control growth was was excluded from the variables included in the final quantitative model.

Figure S1: Observed scores on longitudinal predictors throughout adolescence

Table S3: Fit indices for growth models

| Task | χ^2^ | CFI | TLI | RMSEA |
| --- | --- | --- | --- | --- |
| School attachment | *p*= .15 | 0.999 | 0.997 | 0.024 |
| School commitment | *p*<.05 | 0.991 | 0.989 | 0.036 |
| Parental control | *p*=.25 | 1.000 | 0.999 | 0.013 |
| Street | *p < .05* | 0.994 | 0.992 | 0.032 |
| Park | *p*= .26 | 0.998 | 0.996 | 0.012 |

Fit indices Ideal fit

Chi-square test statistic (χ^2^) Nonsignificant *p*-value

Tucker-Lewis index (TLI) 1

Incremental fit index (IFI) 1

Root-mean-square error of approximation (RMSEA) < 0.05

Table S4: Baseline models for BYDS longitudinal variables

|  | School Attachment | School Commitment | Parental Control | Street | Park |
| --- | --- | --- | --- | --- | --- |
| Intercept | 15.80* (0.14) | 13.31* (0.06) | 13.52* (0.12) | 1.71* (0.03) | 1.05* (0.03) |
| Slope | 0.02 (0.14) | -0.23* (0.05) | -0.17 (0.13) | 0.11* (0.03) | -0.18* (0.02) |
| Quadratic | 0.10* (0.04) |  | -0.08* (0.04) | -0.05* (0.01) | 0.01 (0.01) |
| Variance (intercept) | 19.98* (2.20) | 5.85* (0.34) | 12.28* (1.76) | 1.19* (0.05) | 0.58* (0.07) |
| Variance (slope) | 5.07 (2.66) | 3.80* (0.28) | 0.69 (2.36) | 0.35* (0.04) | 0.28* (0.06) |
| Variance (quadratic) | 0.49* (0.19) |  | 0.25 (0.18) | 0.02* (0.00) | 0.02* (0.00) |
| R (int. slope) | -0.39 (2.35) | -2.82* (0.26) | 2.86 (1.96) | -0.12* (0.02) | -0.19* (0.06) |

Note.

* = *p*< .05

Intercept is the estimated mean score at wave 1 or 2.

Slope loadings show average slope tangent of the line when time equals zero (time at intercept) for school attachment, parental control, street and park. For the school commitment (freed loading) model, the slope represents the mean change between the first and last time point

**Integration analyses – Figure S2**

Figure S2 shows the three phases of the analysis: 1) Latent class analysis of drug use (quantitative); 2) Interviews with drug users (qualitative); 3) Regressions to identify risk factors and outcomes (qualitative). Two integration points were built into the design to maximise the benefits of the study’s mixed methods approach.

The first integration point fell between Phases 1 and 2 and served the purpose of allowing the latent class results to inform the qualitative analysis sampling frame. In Phase 2, participants were purposely sampled from 4 groups (Alcohol; Alcohol & Tobacco; Alcohol, Tobacco & Cannabis; Polydrug) who varied in terms of their NPS-use pattern; the rationale being to use groupings that would help us to distinguish between factors related to NPS use versus those attributable to other drug use. Preliminary regression analyses to identify potential risk factors were also completed in Phase 2 – this meant that highlighted risk factors could be focused on during the interviews.

Further integration analysis took place between Phases 2 & 3. The BYDS longitudinal datasets were screened by qualitative and quantitative team members for variables to use in the Phase 3 quantitative models focusing on risk factors and outcomes related to drug use. BYDS variables were aligned with the risk factors that emerged during Phase 2; node frequency data were used during this process (i.e. risk factors with the highest node frequencies were selected for inclusion in the models). The alignment process was dynamic and iterative in nature and continued until the team felt that optimal alignment had been achieved.

Figure S2: Design of study

Phase

Integration points

1) **Qualitative –> quantitative:** *informed*

*a) Selection of risk factors in quantitative models*

*b) Selection of confounding variables in models predicting adult outcome*

1) **Quantitative –> qualitative** *informed:*

*a) Interview schedules (preliminary regressions)*

*b) Qualitative sampling frame*

3) Regressions to identify risk factors and outcomes

2) Interviews with drug users

1) Latent class analysis of drug use

1. Bollen KA, Curran PJ. Latent curve models: A structural equation perspective: John Wiley & Sons; 2006.
